# Potential diagnostic value of quantitative superb microvascular imaging in premalignant and malignant cervical lesions

**DOI:** 10.3389/fonc.2023.1250842

**Published:** 2023-08-25

**Authors:** Yi Zhu, Yixin Tang, Zhuolin Jiang, Jie Zhang, Shijun Jia, Yanjie Li, Xinyi Luo, Tomoyasu Kato, Guonan Zhang

**Affiliations:** ^1^Outpatient Department (Ultrasound), Sichuan Clinical Research Center for Cancer, Sichuan Cancer Hospital & Institute, Sichuan Cancer Center, Affiliated Cancer Hospital of University of Electronic Science and Technology of China (UESTC), Chengdu, China; ^2^Department of Gynecology, National Cancer Center Hospital, Tokyo, Japan; ^3^Department of Ultrasound, Suining Central Hospital, Suining, China; ^4^Graduate School, Chengdu Medical College, Chengdu, China; ^5^Department Gynecological Oncology, Sichuan Clinical Research Center for Cancer, Sichuan Cancer Hospital & Institute, Sichuan Cancer Center, Affiliated Cancer Hospital of University of Electronic Science and Technology of China (UESTC), Chengdu, China; ^6^Department Pathology, Sichuan Clinical Research Center for Cancer, Sichuan Cancer Hospital & Institute, Sichuan Cancer Center, Affiliated Cancer Hospital of University of Electronic Science and Technology of China (UESTC), Chengdu, China

**Keywords:** cervical cancer, cervical intraepithelial neoplasms, superb microvascular imaging, vascularity index, transvaginal ultrasound

## Abstract

**Objective:**

The purpose of this study was to assess the diagnostic efficacy of the vascular index (VI) on superb microvascular imaging (SMI) in distinguishing normal uterine cervical epithelium, high-grade cervical intraepithelial neoplasia (CIN), and cervical cancer.

**Methods:**

The retrospective study included women with pathology-confirmed CIN or cervical cancer, who underwent transvaginal ultrasound and SMI between April 2021 and October 2022. The SIM manifestations of normal cervix and cervical lesions were reviewed. SIM were measured and converted into vascular index (VI) which compared between cervical lesions and control groups. We have retrospectively compared ultrasound features of cervical lesions and characteristics of patients. Measurement reliability was evaluated by intra class correlation coefficient (ICC).

**Results:**

A total of 235 consecutive females were enrolled, comprising 38 with high-grade CIN, 96 with cervical cancer, and 101 with a normal uterine cervix. The microvascular architecture exhibited significant variations between premalignant and malignant cervical lesions. Branch-like patterns were predominantly observed in high-grade CIN, while crab claw-like and fireball-like patterns were more commonly associated with cervical cancer. The median VI of cervical cancer (34.7 ± 10.3) was significantly higher than that of high-grade CIN (17.6 ± 4.2) (P < 0.001). Moreover, the VI values of cervical cancer differed significantly among different FIGO stages and pathological types (P < 0.001 and P = 0.003, respectively). The VI demonstrated superior diagnostic performance for cervical lesions compared to vascular patterns (AUC = 0.974 and 0.969, respectively). Using a cut-off value of 25.5, the VI yielded a sensitivity of 82.3% and a specificity of 99.3% for cervical lesion detection.

**Conclusions:**

The SMI parameter (VI) exhibited a significantly higher value in cervical cancer compared to high-grade CIN, with a high level of agreement among observers. These findings suggest that quantitative SMI holds promise as an imaging technique for the detection and characterization of cervical lesions.

## Introduction

Cervical cancer ranks as the fourth most prevalent malignant tumor in women, presenting a significant health concern (1). Annually, there are approximately 604,000 new cases of cervical cancer and 342,000 global fatalities associated with the disease, as reported by the Chinese Center for Disease Control and Prevention (2). The etiology of cervical cancer is well-established, with the disease being closely linked to persistent human papillomavirus (HPV) infection (3, 4). Cervical intraepithelial neoplasia (CIN) is a precancerous lesion that exhibits a strong association with cervical cancer. To ensure consistent screening, diagnosis, treatment, and management of cervical precancerous lesions for early detection and intervention, as well as to prevent the onset of cervical cancer, a standardized “three-step screening” protocol has been implemented globally. This protocol involves cervical cytology and HPV testing as the initial step, followed by colposcopy as the second step, and histopathology as the third step. Timely identification and treatment of CIN can significantly reduce the incidence and mortality rates of cervical cancer (5).

Numerous previous studies have consistently demonstrated a close association between tumor development and the formation of new blood vessels (6–8). The origin and abundance of blood supply within a tumor partly reflect its origin, invasion extent, and growth rate. In other words, the microvascular architecture of a tumor serves as a representation of the tumor itself. Recognizing the significance of imaging in cervical cancer staging, the International Federation of Gynecology and Obstetrics (FIGO) officially incorporated imaging into the new staging system in 2018. Consequently, research efforts have increasingly focused on utilizing ultrasound to visualize the blood supply, vascular distribution, and growth of cervical lesions, aiming to facilitate the differentiation between CIN and cervical cancer.

Color Doppler ultrasound is widely used to evaluate tumor angiogenesis in premalignant and malignant cervical lesions (9, 10). Power Doppler (PD) is a useful imaging technique for tumor blood flow, which has the advantages of independent relative angle and velocity, large dynamic range and high sensitivity (11, 12). However, the fine vessels of cervical lesions may not be detected in color or power Doppler ultrasound due to low speed and artifacts. With the emergence of ultrasound contrast agents, a number of clinical studies have reported the application prospect of contrast-enhanced ultrasound (CEUS) in the diagnosis of cervical cancer. The ultrasound contrast agent only stays in the blood vessels and can directly visualize the slow and low-volume blood flow in the tumor microvessels with a diameter of 20 to 39 μm, thereby assessing the tumor vasculature (13). However, CEUS is an imaging modality that requires an interventional procedure with the injection of contrast material.

Superb microvascular imaging (SMI) is an innovative ultrasound image processing technique that incorporates advanced clutter suppression technology. It effectively distinguishes the noise generated by blood flow and tissue motion, employing adaptive computational methods to display accurate blood flow data (14). In comparison to conventional two-dimensional color Doppler ultrasound, SMI has the ability to detect microvascular and low-velocity blood flow, enabling visual assessment of blood perfusion as well as the distribution and density of blood vessels (15–17). SMI serves as a noninvasive approach for monitoring blood flow and displaying vascular distribution, offering advantages in terms of speed, cost-effectiveness, and repeatability. Furthermore, SMI allows for quantitative analysis through a unique parameter known as the vascular index (VI), which is correlated with tumor microvascular density (18, 19). Therefore, qualitative and quantitative investigations of the blood supply source and microvascular architecture of CIN and cervical cancer using SMI have the potential to contribute to accurate diagnosis and staging.

Currently, SMI has demonstrated successful applications in diagnosing various neoplastic lesions (20–24). However, there is a paucity of reports regarding the utilization of this innovative ultrasound technology in diagnosing cervical lesions. Therefore, the objective of this study is to assess the potential value of quantitative SMI as a tool to aid in the identification of CIN and cervical cancer.

## Materials and methods

### Patients

**Figure 1:**
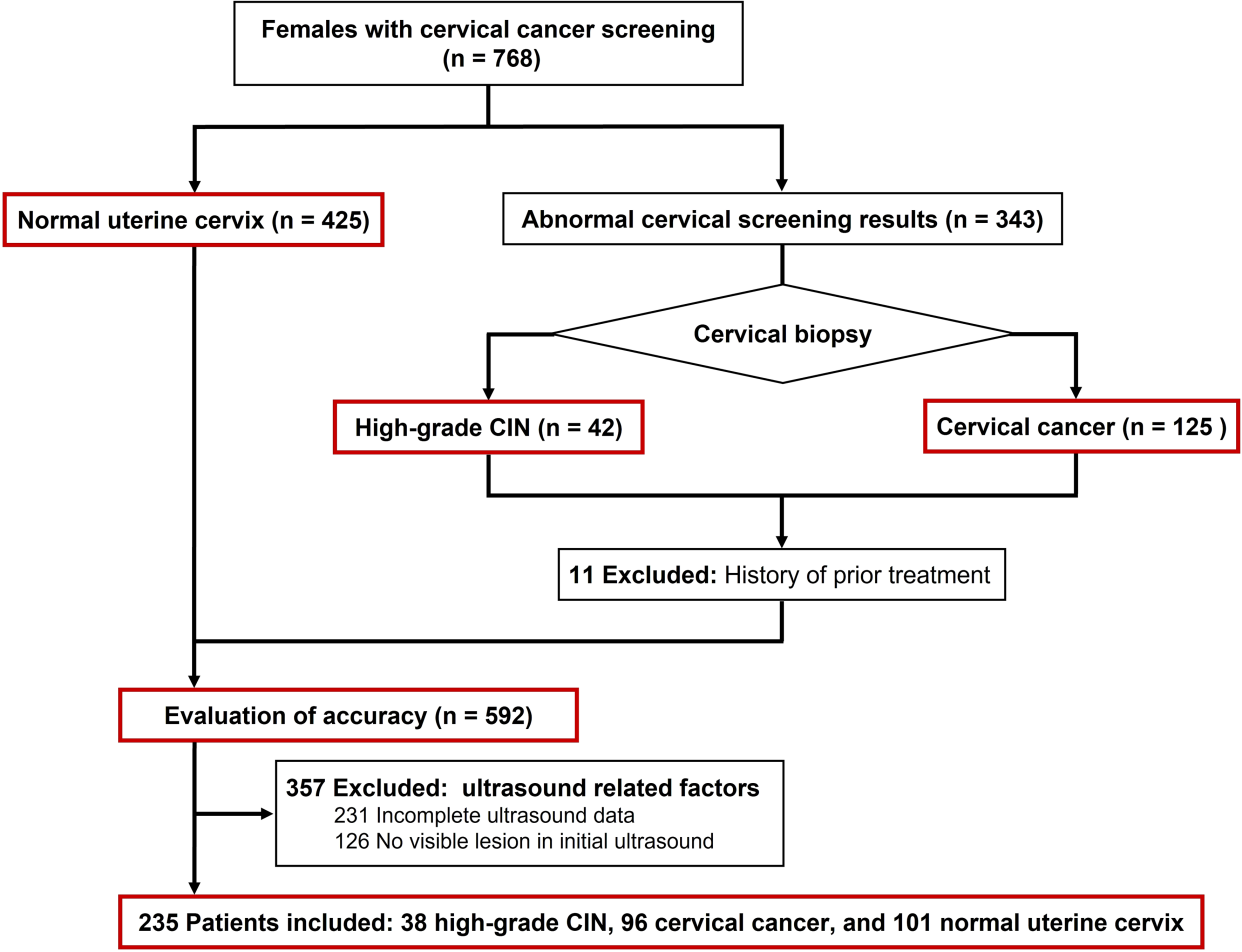
Flowchart of patient enrollment.

This retrospective study was conducted following the review and approval of the institutional ethics committee of Sichuan Cancer Hospital (Approval No. SCCHEC-02-2022-007). The requirement for obtaining written informed consent from the patients was waived. The study included a total of 134 patients with cervical lesions, with a mean age of 48.6 years (range: 28 - 75 years), during the period from April 2021 to October 2022. All patients underwent real-time transvaginal ultrasound (TVUS) and SMI analysis, followed by histopathological diagnosis through multiple punch biopsy or surgery. The staging of patients with cervical cancer was performed based on the FIGO criteria (25). Patients who had previously received treatment, had incomplete medical records, or had inadequate ultrasound imaging were excluded from the study (**Figure 1**). Additionally, a control group comprising 101 cases with normal cervical cytology was selected, with a mean age of 47.5 years (range: 25 - 87 years).

### Transvaginal ultrasound examination

All real-time TVUS examinations were conducted using the Aplio i800 US system (Canon Medical Systems, Tokyo, Japan), employing a multifrequency linear 3 - 11 MHz endovaginal transducer. The examinations were performed by a team of three fellows with more than 8 years of experience in ultrasound imaging of gynecological oncology, who were unaware of pathology results. Throughout the study, the SMI analyses were consistently acquired with the same settings: a depth of 8.5 cm, a focal zone of 3.5, a Doppler frequency of 5.8 MHz, a color gain of 43, and a frame rate exceeding 50 fps (26). These settings were maintained to ensure accurate and comparable quantitative ultrasound measurements. Based on the SMI images, the vascular distributions and patterns were classified into four types: (I) line-like, characterized by few regular lines or spots of flow signals within the uterine cervix; (II) branch-like, exhibiting flow signals within several thick branches in the uterine cervix; (III) crab claw-like, featuring local thick and twisted branched vessels within the lesions; and (IV) fireball-like, displaying uneven thickness and twisted vessel masses resembling fireballs within the lesions. Additionally, the lesion boundary in the region with the strongest blood flow signals on the saved SMI video clips was manually delineated to obtain VI values. The delineation process was performed three times and the results were averaged.

### Statistical analysis

Data management and analysis were conducted using SPSS Version 26.0 (SPSS Inc., Chicago, IL, USA). Continuous variables were analyzed using the Mann-Whitney test, while categorical variables were analyzed using Fisher’s exact test. To evaluate the diagnostic value of SMI for cervical cancer, receiver operating characteristic (ROC) curves and area under the curve (AUC) were employed. The interclass correlation coefficient (ICC) was calculated to assess interobserver reproducibility. A p-value of less than 0.05 was considered statistically significant.

## Results

### Patient demographics and tumor characteristics

**Table 1** showed the clinic and demographic characteristics of the population divided into the three groups. The study included a total of 134 patients with cervical lesions. The pathological results indicated that among these patients, 38 cases were diagnosed with high-grade CIN, with a mean age of 46.2 years (range: 28 - 75 years), while 96 cases were diagnosed with cervical cancer, with a mean age of 49.6 years (range: 33 - 72 years). Among the cervical cancer cases, 69 were classified as squamous cell carcinoma, 21 as adenocarcinoma, and 6 as small cell neuroendocrine carcinoma. Based on the FIGO staging system, 20 cases were categorized as stage I, 23 as stage II, 36 as stage III, and 17 as stage IV.

**Table 1:** The clinic and demographic characteristics of the investigated population.

	Normal uterine cervix(n = 101)	High-grad CIN(n = 38)	Cervical cancer(n = 96)	P
Age, y*	47.5 ± 12.5	46.2 ± 11.1	49.6 ± 9.4	0.195
BMI, kg/m^2^*	23.2 ± 3.9	23.0 ± 3.6	22.7 ± 4.0	0.567
Smoking habit				0.698
Yes	3	2	5	
No	98	36	91	
Personal/family history of tumors				
Yes	5	0	2	0.095
No	96	38	94	

*Results are presented as Mean ± SD (Minimum - Maximum). BMI, Body mass index. CIN, cervical intraepithelial neoplasia.

### Vascular patterns in SMI image

**Figure 2:**
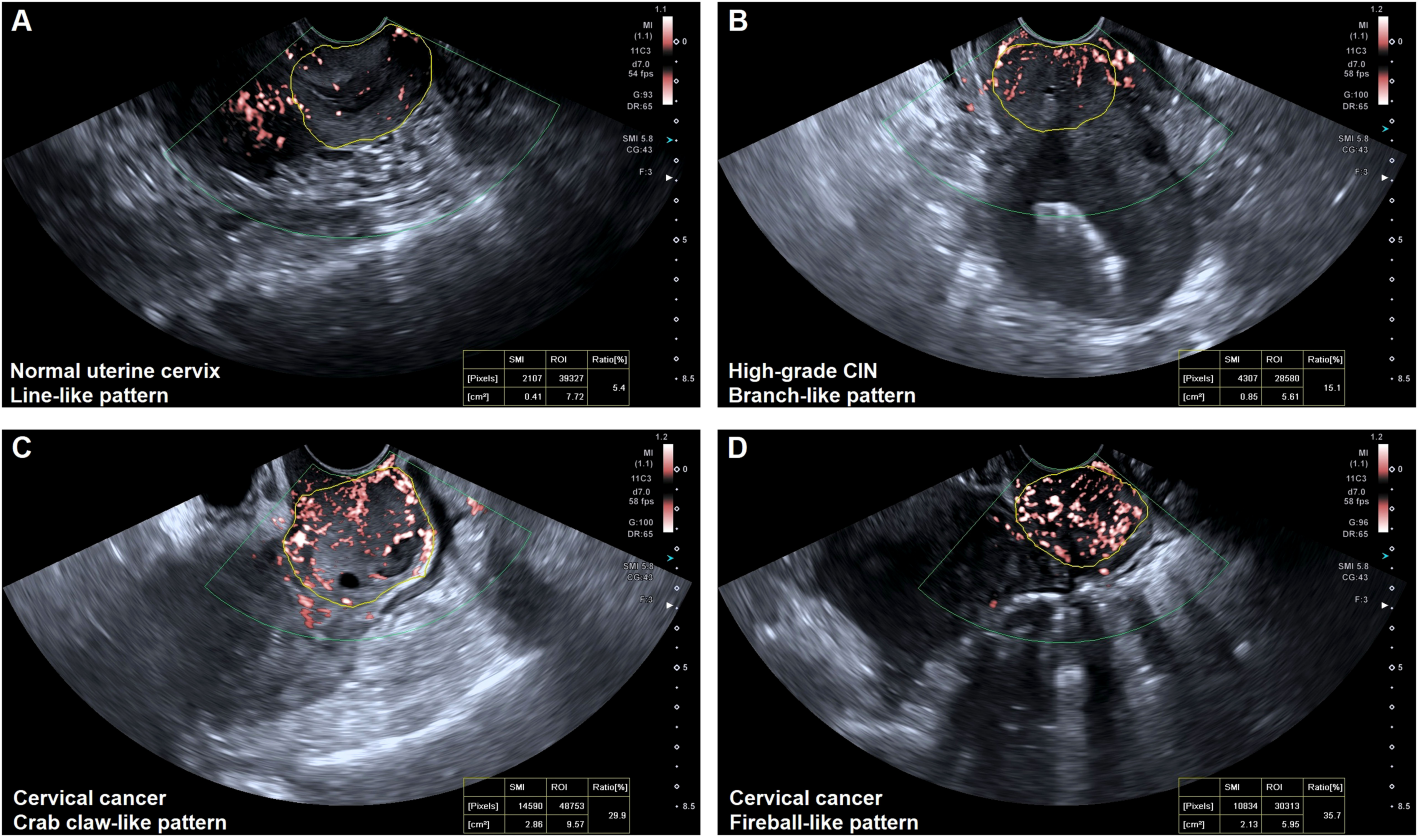
Vascular features of the normal uterine cervix and cervical lesions. **(A)** Line-like pattern: SMI imaging revealed a few regular lines and spots of flow signals within the normal uterine cervix. **(B)** Branch-like pattern: SMI imaging showed several thick branches with flow signals within the uterine cervix (high-grade CIN). **(C)** Crab claw-like pattern: SMI imaging showed local thick and twisted branched vessels within cervical cancer (FIGO stage IIIa). **(D)** Fireball-like pattern: SMI imaging revealed a mass of fire-like spherical vessels within cervical cancer (FIGO stage IVa).

The blood flow was detected in all cases (100%) of normal uterine cervix, high-grade CIN, and cervical cancer using SMI. However, there were significant differences in the vascular patterns between premalignant and malignant cervical lesions. The vascular features of cervical lesions were classified into four patterns: line-like, branch-like, crab claw-like, and fireball-like (**Figure 2**). Among the normal uterine cervix cases, 96 (95.0%) exhibited a line-like pattern, while only 5 (5.0%) showed branch-like patterns. In contrast, a larger proportion of high-grade CIN (73.7%) displayed branch-like patterns, while the majority of cervical cancer cases (86.5%) exhibited crab claw-like or fireball-like patterns (P < 0.001) (**Table 2**). Notably, 65.0% (13/20) of stage I cervical cancer cases had a branch-like pattern, similar to high-grade CIN (P = 0.084). When crab claw-like and fireball patterns were used as diagnostic criteria for cervical cancer, SMI demonstrated a sensitivity of 86.5%, specificity of 78.9%, positive predictive value (PPV) of 91.2%, negative predictive value (NPV) of 92.9%, and accuracy of 84.3%.

**Table 2:** Vascular features of cervical lesions on SMI.

Vascular features	Normal uterine cervix (n = 101)	High-grad CIN (n = 38)	Cervical cancer (n = 96)
**Line-like**	96 (95.0)	2 (5.3)	0
**Branch-like**	5 (5.0)	28 (73.7)	13 (13.5)
**Crab claw-like**	0	8 (21.0)	24 (25.0)
**Fireball-like**	0	0	59 (61.5)

Results are presented as n (%). SMI, superb microvascular imaging. CIN, cervical intraepithelial neoplasia.

### Quantitative SMI analysis of cervical lesions

The mean vascular index (VI) of cervical cancer (34.7 ± 10.3) was higher compared to high-grade CIN (17.6 ± 4.2) and normal uterine cervix (4.6 ± 2.9) (P < 0.001). **Table 3** provides an overview of the characteristics and mean VI values of cervical cancer. The mean VI values of advanced cervical cancer (stage II to IV) showed a gradual increase with the progression of FIGO stage (P = 0.002) and were significantly higher than those of stage I cervical cancer (P < 0.001). Conversely, the mean VI of stage I cervical cancer (20.6 ± 7.7) was slightly higher than that of high-grade CIN, but the difference was not statistically significant (P = 0.125). Neuroendocrine small cell carcinoma of the cervix exhibited poor blood supply, and its VI was significantly lower than that of squamous cell carcinoma and adenocarcinoma. In stage I cervical cancer, the mean VI of cervical squamous cell carcinoma (16.8 ± 2.6) was lower compared to adenocarcinoma (32.2 ± 5.6) (P = 0.003).

**Table 3:** Characteristics and the mean VI value of cervical cancer.

Characteristics	No. of patients (%)	Vascular index	P
FIGO stage			< 0.001
**I**	20 (20.8)	20.6 ± 7.7	
**II**	23 (24.0)	35.6 ± 6.8	
**III**	36 (37.5)	37.9 ± 5.9	
**IV**	17 (17.7)	43.6 ± 8.0	
Histologic type			
**SCC**	69 (71.9)	35.64 ± 10.9	0.003
**AC**	21 (21.9)	35.7 ± 5.7	
**NSCC**	6 (6.2)	21.1 ± 5.7	

Data are presented as mean ± standard deviation. FIGO, the International Federation of Gynecology and Obstetrics. SCC, squamous cell carcinoma. AC, adenocarcinoma. NSCC, neuroendocrine small cell carcinoma.

### Diagnostic performances of vascular patterns and the VI of SMI

**Figure 3:**
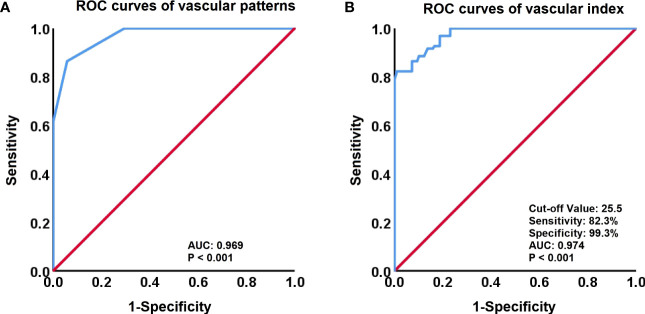
Receiver-operating characteristic (ROC) curves for vascular patterns and Vascular Index (VI). **(A)** ROC curves of vascular patterns in the discrimination between High-Grade CIN and Cervical Cancer. **(B)** ROC curves of the VI (cut-off value, 25.5) to identify High-Grade CIN and Cervical Cancer.

To differentiate between premalignant and malignant cervical lesions, an ROC analysis was performed for both vascular patterns and quantitative SMI (VI) (**Figure 3**). Both vascular patterns and VI demonstrated the ability to distinguish cervical lesions, with an AUC of 0.969 and 0.974, respectively. The optimal cut-off value for VI in the differential diagnosis was determined to be 25.5. Moreover, VI exhibited superior diagnostic performance for cervical cancer compared to vascular patterns, with a sensitivity of 82.3% and specificity of 99.3%.

### Reproducibility analysis

The intraclass correlation coefficient (ICC) for measuring the VI was 0.913 (95% CI, 0.879 - 0.939; P < 0.001), indicating a high level of agreement and almost perfect consistency.

## Discussion

SMI, initially introduced in 2011 by Toshiba Medical Systems (now Canon Medical Systems), is an innovative ultrasound technology designed to visualize and analyze blood flow within the microvasculature, which comprises the small blood vessels responsible for supplying blood to tissues and organs. SMI offers several advantages, including high sensitivity, high resolution, and reduced motion artifacts, enabling the visualization of detailed microvascular structures. By evaluating the number and distribution of microvessels, SMI addresses the limitations of color Doppler flow imaging (CDFI) and power Doppler imaging (PDI), allowing for a more objective assessment of tumor blood supply (27–29). Our study’s findings demonstrated that SMI exhibited a high sensitivity (86.5%) in diagnosing cervical cancer, effectively identifying patients with cervical cancer, particularly those in intermediate and advanced stages. Moreover, SMI exhibited a high negative predictive value (NPV) of 92.9%, further highlighting its potential as a screening tool for cervical lesions. It is important to note that the results of this study are novel and have not been previously reported in the literature.

The blood supply to the uterine body is primarily through the ascending branch of the uterine artery, while the cervix receives its blood supply mainly from the descending branch of the uterine artery (cervical branch). The descending branch gives rise to branches that extend to the vaginal vault and form a vascular plexus running on both sides of the vagina, along with the ascending branch of the vaginal artery, which supplies the middle part of the vagina. Normally, the blood flow in the uterine body is significantly greater than that in the cervix. As a result, SMI typically shows sparse punctate or short rod-shaped blood flow in the majority of the normal uterine cervix. When a lesion appears in the uterine cervix, the development of the descending branch of the uterine artery is initiated. The microvascular architecture in the cervical region undergoes changes that are closely related to the nature of the lesion. In patients with high-grade CIN, the local cervical blood vessels show increased numbers and thickening compared to a normal cervix. In cases of cervical cancer, the rapid growth of the lesion requires a significant amount of blood flow, leading to the generation of numerous new blood vessels. However, due to the absence of smooth muscle neovascularization walls, the blood flow rate becomes faster, resulting in a “sifting phenomenon” where the blood flow is redistributed from the uterine artery supplying the upper and lower branches. Consequently, the descending branch of the uterine artery becomes thicker, leading to a significant increase in blood supply to the cervical lesion. SMI imaging in patients with cervical cancer reveals increased, thickened, dense, and disordered blood vessels at the tumor site, characterized by abundant blood flow. Some of these vessels exhibit a “vessel pool” structure, resembling a “fireball-like” appearance. Our study observed that as cervical lesions progress and worsen, the distribution of blood vessels transitions from uniform and sparse to disorganized and numerous. Cervical SMI provides a more objective reflection of the extent of cervical lesions, thereby serving as a valuable reference for diagnosis and differential diagnosis.

One of the key parameters used to quantify blood flow in SMI is the Vascular Index (VI). VI is a numerical measurement that represents the amount of blood flow within a specific region of interest. It is calculated by analyzing the amplitude and frequency of the Doppler signal generated by the moving blood cells within the vessels. A higher VI value indicates a greater amount of blood flow within the region of interest. VI can be used to evaluate microvascular perfusion in various organs and tissues (30, 31). In our study, we observed that the VI value increased with the severity of the lesion, with significantly lower VI values in the normal cervical group compared to the high-grade CIN group and the cervical cancer group (P < 0.001). Furthermore, the VI value was significantly higher in advanced cervical cancer (stage II-IV) compared to stage I cervical cancer and high-grade CIN, indicating a significant increase in the number of blood vessels in cervical cancer with advancing stages. This finding is consistent with the understanding that neovascularization is a necessary condition for the growth of malignant tumors, and it can help in the identification and staging of cervical lesions. However, distinguishing stage I cervical cancer from high-grade CIN based on tumor angiogenesis alone can be challenging, as both demonstrate similar patterns of angiogenesis. Lesions with poor blood flow may be less responsive to radiotherapy and chemotherapy, resulting in a poor treatment response (32, 33). Our study also revealed that the VI of cervical neuroendocrine small cell carcinoma was significantly lower than that of cervical squamous cell carcinoma and adenocarcinoma, which may partly explain the poor prognosis associated with neuroendocrine small cell carcinoma. Additionally, early-stage cervical adenocarcinoma exhibited a higher richness of blood flow compared to squamous cell carcinoma, and the blood flow richness of squamous cell carcinoma increased with advancing stages, which could partly explain the poorer prognosis observed in adenocarcinoma (34). However, further research is needed to verify the correlation between the degree of blood supply and the pathological types of cervical cancer.

Our study has several limitations that should be acknowledged. Firstly, it was conducted at a single medical center and involved a relatively small number of cases, particularly with limited enrollment of high-grade CIN and stage I cervical cancer cases. This may introduce some bias and limit the generalizability of our findings. Secondly, there is a possibility of observer variability in assessing the VI of lesions. However, it is important to note that our study demonstrated excellent interobserver reproducibility in SMI quantitative analysis. VI is a highly reproducible and objective quantitative parameter of SMI. Thirdly, in our study, we measured VI in the region with the strongest blood flow signal within the preserved video clips (19, 28). However, it is possible that these selected images may not fully represent the overall blood supply of the entire tumor. It was reported that smart three-dimensional (3D) SMI was helpful to visualize the microvascular architecture of the overall tumor or organ, to determine the optimal two-dimensional (2D) SMI plane with the most abundant vasculature to guide VI quantitative measurements of lesions (14). But 3D-SMI is not yet feasible for TVUS. In addition, our study lacked validation of vascular distribution based on pathological findings, which will present results in our future SMI series. Finally, it is important to acknowledge that SMI has limitations in eliminating artifacts caused by breathing or visceral movement, and it may sometimes face challenges in accurately distinguishing between blood vessels (arteries or veins) within the microvascular region.

## Conclusion

In summary, our study highlights that the VI value of cervical cancer is significantly higher compared to cervical precancerous lesions (high-grade CIN), demonstrating its potential as a reliable and effective differential diagnostic tool. Additionally, in another study, we have previously reported the utility of SMI quantitative analysis in monitoring tumor response to chemoradiotherapy in locally advanced cervical cancer (26). These findings collectively emphasize the value of quantitative SMI in accurately quantifying microvascular blood flow and evaluating perfusion in premalignant and malignant cervical lesions. Such assessments can aid in predicting tumor aggressiveness and informing treatment decisions. However, it is important to note that future studies with larger sample sizes are necessary to further validate and reinforce our findings.

## Author’s note

We would like to express our gratitude to Professor Tomoyasu Kato from the Department of Gynecology at the National Cancer Center Hospital of Japan for his valuable guidance and helpful revisions of the article.

## Data availability statement

The raw data supporting the conclusions of this article will be made available by the authors, without undue reservation.

## Ethics statement

The studies involving humans were approved by the institutional ethics committee of Sichuan Cancer Hospital. The studies were conducted in accordance with the local legislation and institutional requirements. The ethics committee/institutional review board waived the requirement of written informed consent for participation from the participants or the participants’ legal guardians/next of kin because this was a retrospective study. Written informed consent was obtained from the individual(s) for the publication of any potentially identifiable images or data included in this article.

## Author contributions

YZ and GZ conceived, designed, or planned the study. YZ, YT, JZ and SJ provided study patients, imaging and pathological data. ZJ, YL and XL collected or assembled the data. YZ and YT performed or supervised analyses. YZ wrote sections of the initial draft. YZ and GZ provided substantive suggestions for revision or critically reviewed subsequent iterations of the manuscript. TK and GZ provided administrative, technical, or logistic support. YZ and YT contributed equally to this work. All authors reviewed and approved final version of the paper; are accountable for all aspects of the work in ensuring that questions related to the accuracy or integrity of any part of the work are appropriately investigated and resolved.
